# Proteomic Analysis Reveals Dab2 Mediated Receptor Endocytosis Promotes Liver Sinusoidal Endothelial Cell Dedifferentiation

**DOI:** 10.1038/s41598-017-13917-9

**Published:** 2017-10-18

**Authors:** Yuanxiang Lao, Yanyan Li, Yufang Hou, Huahai Chen, Bintao Qiu, Weiran Lin, Aihua Sun, Handong Wei, Ying Jiang, Fuchu He

**Affiliations:** 1 Institute of Basic Medical Sciences, Chinese Academy of Medical Sciences, School of Basic Medicine, Peking Union Medical College, Beijing, China; 2 State Key Laboratory of Proteomics, National Center for Protein Sciences, Beijing, Beijing Proteome Research Center, Beijing Institute of Radiation Medicine, Beijing, China; 3 School of Life Sciences, Tsinghua University, Beijing, China

## Abstract

Sinusoidal dedifferentiation is a complicated process induced by several factors, and exists in early stage of diverse liver diseases. The mechanism of sinusoidal dedifferentiation is poorly unknown. In this study, we established a NaAsO_2_-induced sinusoidal dedifferentiation mice model. Liver sinusoidal endothelial cells were isolated and isobaric tag for relative and absolute quantitation (iTRAQ) based proteomic approach was adopted to globally examine the effects of arsenic on liver sinusoidal endothelial cells (LSECs) during the progression of sinusoidal dedifferentiation. In all, 4205 proteins were identified and quantified by iTRAQ combined with LC-MS/MS analysis, of which 310 proteins were significantly changed in NaAsO_2_ group, compared with the normal control. Validation by western blot showed increased level of clathrin-associated sorting protein Disabled 2 (Dab2) in NaAsO_2_ group, indicating that it may regulate receptor endocytosis, which served as a mechanism to augment intracellular VEGF signaling. Moreover, we found that knockdown of Dab2 reduced the uptake of VEGF in LSECs, furthermore blocking VEGF-mediated LSEC dedifferentiation and angiogenesis.

## Introduction

Liver sinusoidal endothelial cell (LSEC) is a type of liver specific microvascular cell, which characterizes unique phenotype and function. In normal liver, differentiated LSECs form capillaries of microvasculature and facilitate filtration by fenestrae as a selectively permeable barrier between liver parenchyma and sinusoid^1^. Upon liver injury (e.g., fibrosis^2–4^, hepatitis^5,6^, alcoholic liver injury^7^ and arsenic exposing^8^), LSECs loss their highly specialized fenestration and gain an organized basement membrane, which calls LSEC dedifferentiation or capillarization. Although the mechanism of LSEC dedifferentiation has been comprehensively studied, the molecular mechanisms driving dedifferentiation have not been fully elucidated. So far, there are few ideal models to study the molecular mechanisms of LSEC dedifferentiation *in vivo*, as most models cause cirrhotic fibrosis simultaneously, obfuscating the real issues of LSEC dedifferentiation. However, Straub *et al*.^9^ tested the effects of sodium arsenite (NaAsO_2_) on dedifferentiated LSECs and proved that NaAsO_2_ induced LSEC dedifferentiation without fibrosis initiation. Therefore, NaAsO_2_-induced LSEC dedifferentiation mice model could be applied to study LSEC dedifferentiation mechanisms.

In this study, we used NaAsO_2_ in drinking water of mice for 5 weeks as the early injury phase to induce LSEC dedifferentiation, comparing with normal mice. Then LSECs from these two groups were isolated and lysed. Isobaric tags for relative and absolute quantification (iTRAQ) coupled with LC-MS/MS was used for relative quantification of proteins *in vivo*, based on a more powerful and sensitive proteomic method than traditional approaches, especially quantifying low-abundance proteins^10–12^. Protein identification and quantification was accurately performed using Protein Pilot Software with specifically developed algorithms.

For our experiments, iTRAQ-labeled LSECs in NaAsO_2_-induced LSEC dedifferentiation mice model were first used for differentially expressed proteome analysis through Protein Pilot software, and 4205 proteins were identified. Among these, there were 207 up-regulated proteins and 103 down-regulated proteins, respectively. For functional analysis in depth, we found that two significantly increased proteins, disabled homolog 2 (Dab2) and clathrin heavy chain (CLTC), were involved in VEGF receptor endocytosis, serving as a mechanism to induce intracellular receptor signaling upon the stimulation of VEGF signal, which is referred as a regulator leading to angiogenesis^13^.

## Results

### Confirmation of NaAsO_2_-induced LSEC dedifferentiation in mice model

**Figure 1:**
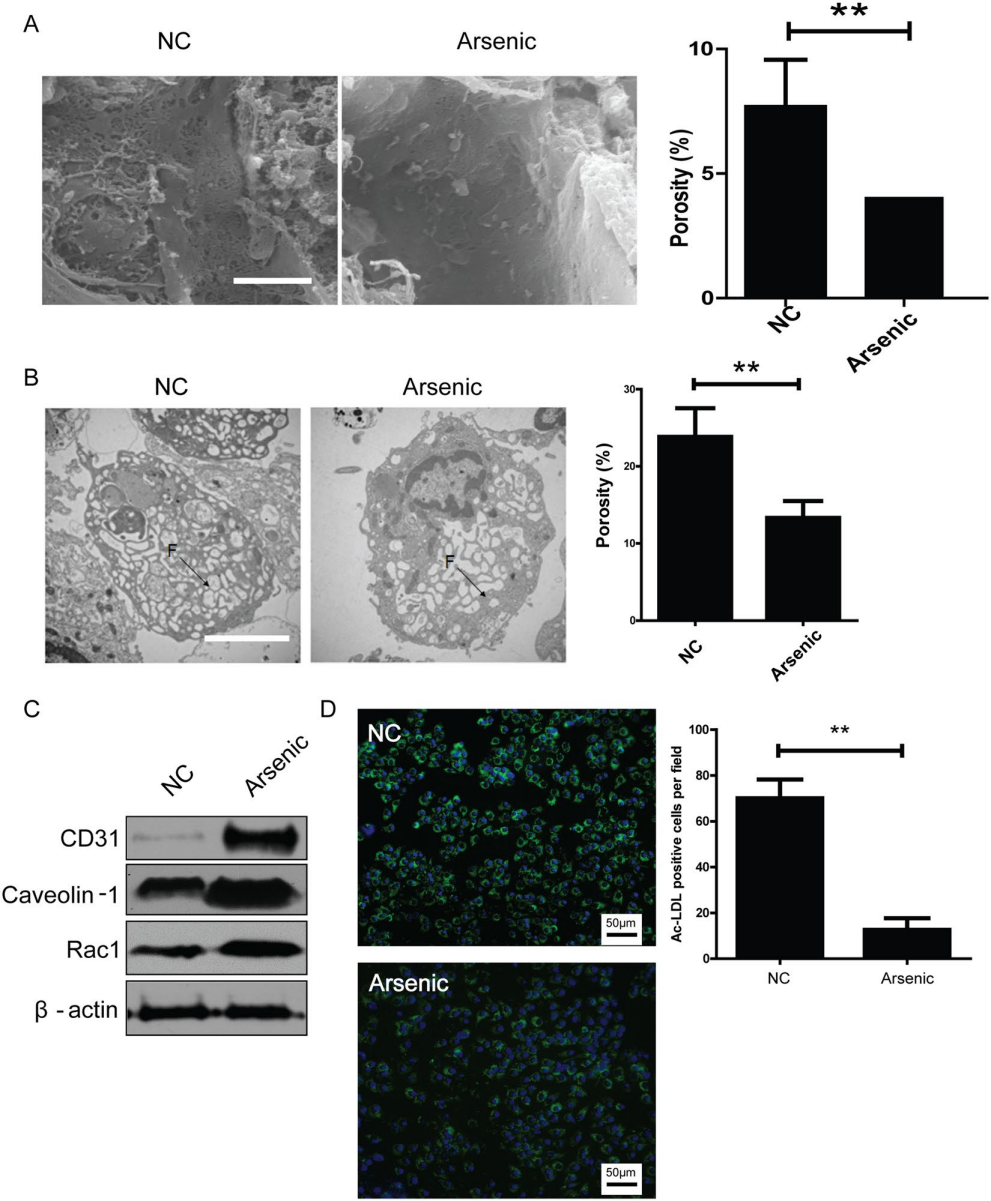
NaAsO_2_-induced LSEC dedifferentiation. (**A**) Representative SEM images of liver sinusoids (n = 5) and quantitative porosity of sinusoidal fenestrae in normal and NaAsO_2_-induced groups. Bar = 1μm. **P < 0.01. (**B**) Representative TEM images of primary LSECs isolated from normal (n = 5) and NaAsO_2_-induced group (n = 5) and quantitative porosity of sinusoidal fenestrae. Bar = 1μm. **P < 0.01. (**C**) The expressions of CD31, Caveoloin-1 and Rac1 in normal and NaAsO_2_-induced groups were analyzed by western blot. All western blot experiments were repeated at least three times. Full-length blots are included in Supplemental Information. (**D**) Uptake of Ac-LDL was analyzed by fluorescence microscopy in primary LSECs isolated from normal and NaAsO_2_-induced groups and cultured overnight. All experiments in (**C**) and (**D**) were repeated at least three times.

0.25 μg/mL NaAsO_2_ in drinking water was administered for 5 weeks in mice to induced LSEC dedifferentiation as described previously^9^. The open fenestrae of liver sinusoids were decreased in arsenic exposed group, compared with the normal counterparts (7.46 ± 0.41% vs 3.56 ± 0.76%) (Fig. 1A). The permeability of isolated LSECs from arsenic exposed group, identified by porosity of cell surface, was also reduced (22.64 ± 5.38% vs 13.35 ± 2.78%) (Fig. 1B). Meanwhile, the expression of LSEC dedifferentiation markers, such as CD31, Caveolin-1 and Rac1, was increased in arsenic exposed group (Fig. 1C). But LSEC differentiation marker, the uptake of acetylated low density lipoprotein (Ac-LDL), was instead reduced in arsenic exposed group (Fig. 1D). These results suggested that 5-week NaAsO_2_ administration successfully induced LSEC dedifferentiation in mice.

### Purity and characterization of primarily isolated LSECs

To generate LSEC proteome, we isolated LSECs from normal and arsenic exposed mice respectively using a modified protocol including two-step collagenase perfusion, centrifugation and magnetic beads sorting, as described previously^14–16^. Purity and viability of LSECs were up to 93.6 ± 1.7% and 88.4 ± 0.5% confirmed by CD146 + F4/80- and 7-AAD + flow cytometry analysis separately, and yield of LSECs was approximately (2.1 ± 0.2) × 10^6^ per mouse, (Supplemental Fig. 1A–C). Nextly, we assessed the quality of primary LSECs to exclude the false positive analysis of flow cytometry. After 8 h culture and extensive washing, LSEC monolayer showed a typical cobblestone, sheet-like appearance (Supplemental Fig. 1D). In liver, Ac-LDL was mainly taken up by LSECs^17^. Therefore, fluorescently labeled Ac-LDL was used to confirm LSEC quality^18^. After overnight culture, above 98% LSECs were labeled by Ac-LDL, according to Ac-LDL endocytosis assay (Supplemental Fig. 1E). These results demonstrated that primary LSECs with high purity and viability were obtained.

### Proteome differential analysis of normal and dedifferentiated LSECs by iTRAQ

To elucidate the molecular mechanisms of LSEC dedifferentiation, quantitative proteomic analysis based on iTRAQ labeling was executed between NaAsO_2_ induced LSEC dedifferentiation mice model and the counterparts. Total 7763 proteins were identified in two independent biological replicates (FDR < 1%). Among these, 54.16% (4205/7763) proteins were shared by these two experiments (Supplemental Fig. 2A and Supplemental Table 1). In addition, linear regression analysis was performed with ln [115/116 ratio] and ln [116/115 ratio] in these two independent experiments to examine the biological reproducibility, and the Pearson correlation coefficient was 0.7181 (P < 0.0001), indicating high biological reproducibility of our experiments. To identify significant up- or down-regulated proteins during LSEC dedifferentiation, the threshold values of 115/116 or 116/115 ratios were ≥1.50 or ≤0.67 (≥1.5-fold) in both two iTRAQ analyses. Accordingly, 207 and 103 proteins were significantly up- or down-regulated, respectively, in dedifferentiated LSECs (Supplemental Tables 2 and 3), suggesting dramatic alterations during LSEC dedifferentiation.

**Figure 2:**
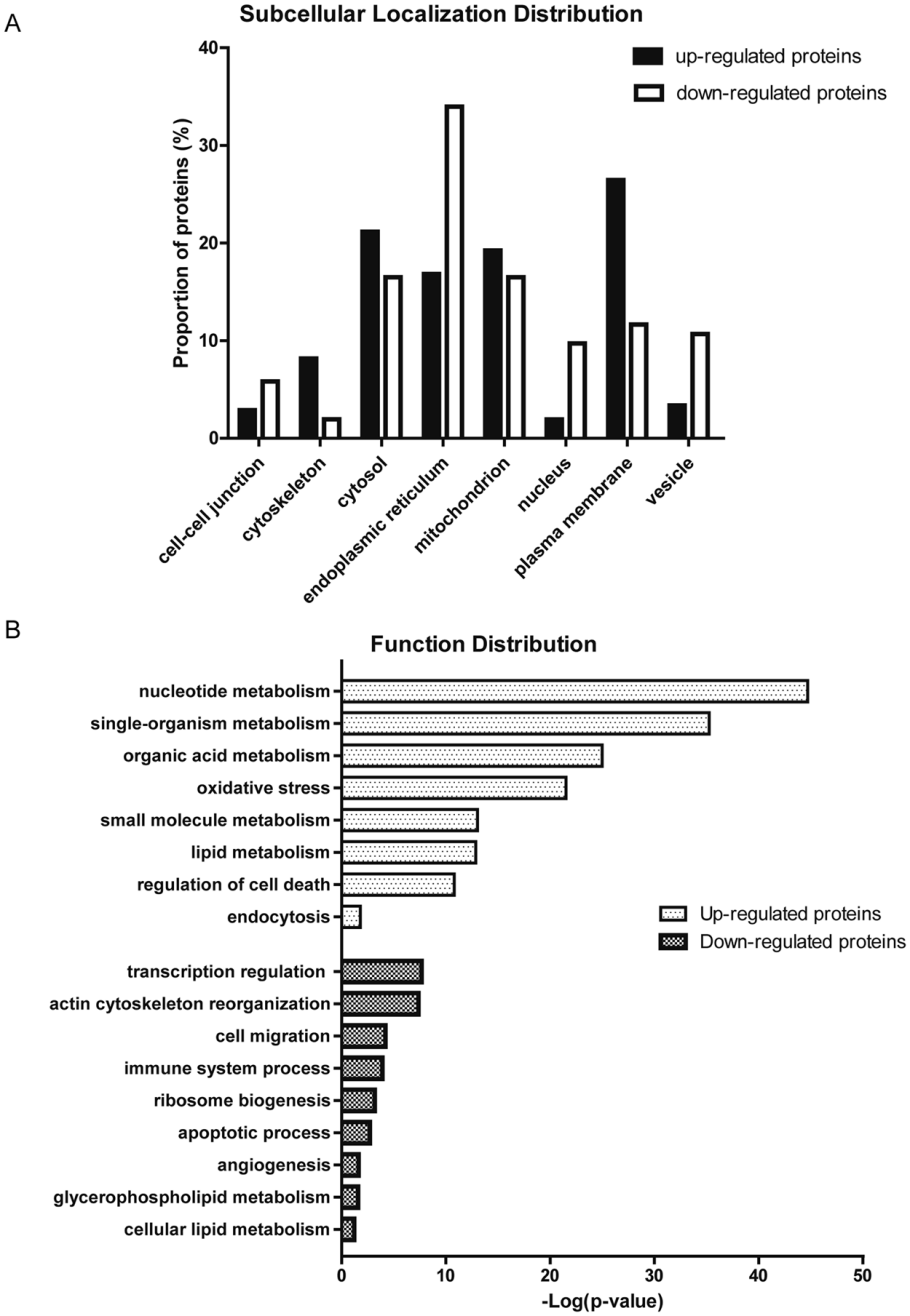
Subcellular localization and biological functions of differential proteins during LSEC dedifferentiation (**A**) Subcellular localization of altered proteins during LSEC dedifferentiation. (**B**) Biological functions of differential proteins during LSEC dedifferentiation. The top biological functions of up-regulated and down-regulated proteins were determined by Gene Ontology and DAVID annotation analysis.

### Bioinformatic analysis of differentially expressed proteins

The 310 differentially expressed proteins were categorized by their cellular component and biological function using Gene Ontology analysis (GO) or DAVID functional annotation. Most of up-regulated proteins were localized in plasma membrane and cytosol, while down-regulated proteins in endoplasmic reticulum and mitochondrion (Fig. 2A), indicating that differentially expressed proteins are significantly devided at the subcellular level. The enriched biological functions of up-regulated proteins were mainly associated with multiple component metabolism (such as nucleotide, single-organism, organic acid, small molecular, lipid, *et al*.), oxidative stress, cell survival and endocytosis (Fig. 2B), showing oxidative stress and energy metabolism are involved in LSEC dedifferentiation process, in accordance with the findings described previously^8,9^. Interestingly, endocytosis was the unique function with top enrichment score, suggesting endocytosis may mediate LSEC dedifferentiation. Meanwhile, proteins associated with transcription regulation, immune system process, ribosome biogenesis, apoptotic process, *et al*., were all down-regulated during LSEC dedifferentiation (Fig. 2B), among which the reduced immune system process might disable defense line against arsenic insult. These findings discovered that endocytosis induced LSEC dedifferentiation for the first time. In addition, LSEC were also found to lose the defense ability against injury insults.

### Experimental Validation of Proteomic analysis in LSECs

**Figure 3:**
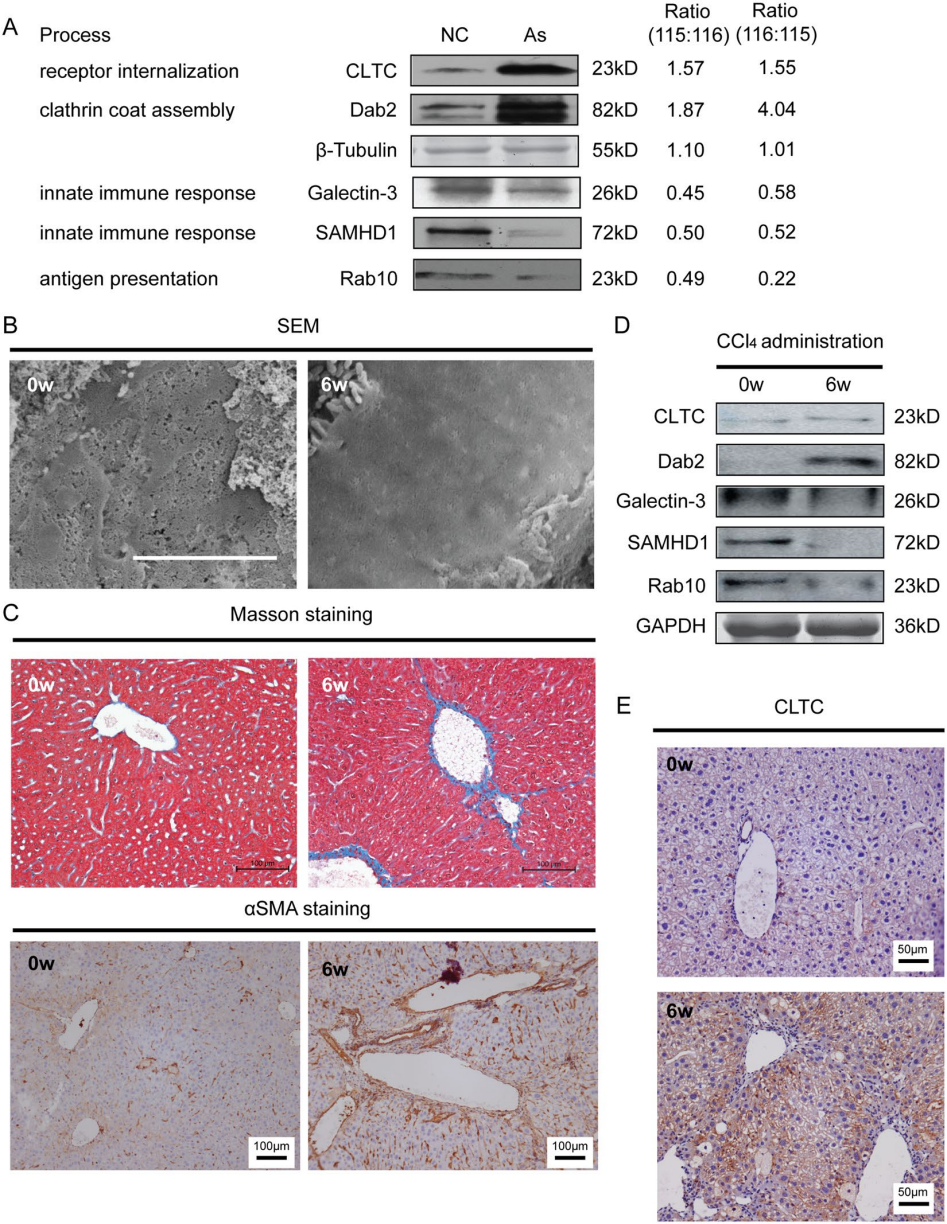
Validation of selected proteins in LSECs from NaAsO_2_-induced LSEC dedifferentiation and CCl_4_-induced liver injury mice model. (**A**) Differential expressions of 2 up-regulated proteins (CLTC, Dab2) and 3 down-regulated proteins (Galectin-3, SAMHD1, Rab-10) were validated by Western blot. The relative ratios of proteins by iTRAQ analysis were shown right. All western blot experiments were repeated at least three times. Full-length blots are included in Supplemental Information. (**B**) Representative SEM images of liver sinusoids (n = 5) and quantitative porosity of sinusoidal fenestrae at 0 and 6^th^ week with CCl_4_ administration. Bar = 1μm. (**C**) Masson’s trichrome staining and αSMA expression in liver at 6^th^ week with CCl_4_ administration (n = 5) were analyzed by immunohistochemistry analysis, Bar = 100μm. (**D**) Western blot analysis of primary LSECs isolated from 0 and 6^th^ week for the levels of CLTC, Dab2, Galectin-3, SAMHD1 and Rab-10 proteins, and GAPDH was used as loading control. All western blot experiments were repeated at least three times. Full-length blots are included in Supplemental Information. (**E**) CLTC expression in liver at normal control mice and mice at 6^th^ week with CCl_4_ administration (n = 5) were analyzed by immunohistochemistry analysis, Bar = 100μm.

Differential expressions of 5 selected proteins were further validated by western blot, focusing on those involved in receptor endocytosis and innate immune response. Compared with the normal LSECs, proteins involved in receptor endocytosis (CLTC) and clathrin coat assembly (Dab2) were significantly up-regulated in arsenic induced dedifferentiated LSECs, whereas three proteins (Galectin-3, SAMHD1, Rab10) related to innate immune response and antigen presentation showed significant down-regulation in dedifferentiated LSECs. The western blot results were in keeping with the iTRAQ data (Fig. 3A).

For further validation, the chronic liver injury mice model induced by carbon tetrachloride (CCl_4_) was established as described previously^19^, generating LSEC dedifferentiation at 6^th^ week after CCl_4_ administration (Fig. 3B). And this model was further verified by Masson trichrome staining and αSMA immunohistochemistry, showing obvious chronic liver injury (Fig. 3C). Differential expression of selected proteins was further evaluated by Western blot in LSECs isolated from *in vivo* model, and confirmed CLTC and Dab 2 were increased at the 6^th^ week after CCl_4_ administration, when LSECs were dedifferentiated and fibrotic septa formed. Galectin-3, SAMHD1, Rab10 showed down-regulation as displayed in Fig. 3D. The expression of CLTC, which is marker of clathrin-coat associated receptor endocytosis, was increased in liver of CCl_4_-treated mice at the 6^th^ week, comparing with normal counterparts (olive given only) (Fig. 3E). These results further proved the expression levels of these proteins in chronic liver injury.

### The biological significance of Dab2 in LSEC dedifferentiation

Expression of clathrin heavy chain 1 (CLTC), an important mediator regulating receptor mediated endocytosis^20^, was up-regulated in dedifferentiated LSECs based on our proteome data and the following protein validation. Meanwhile, disabled homolog 2 (Dab2), a clathrin-associated sorting protein^21^, which mediates clathrin-dependent VEGF receptor endocytosis^13^, was also up-regulated, supporting that VEGF receptor endocytosis may be involved in LSEC dedifferentiation.

**Figure 4:**
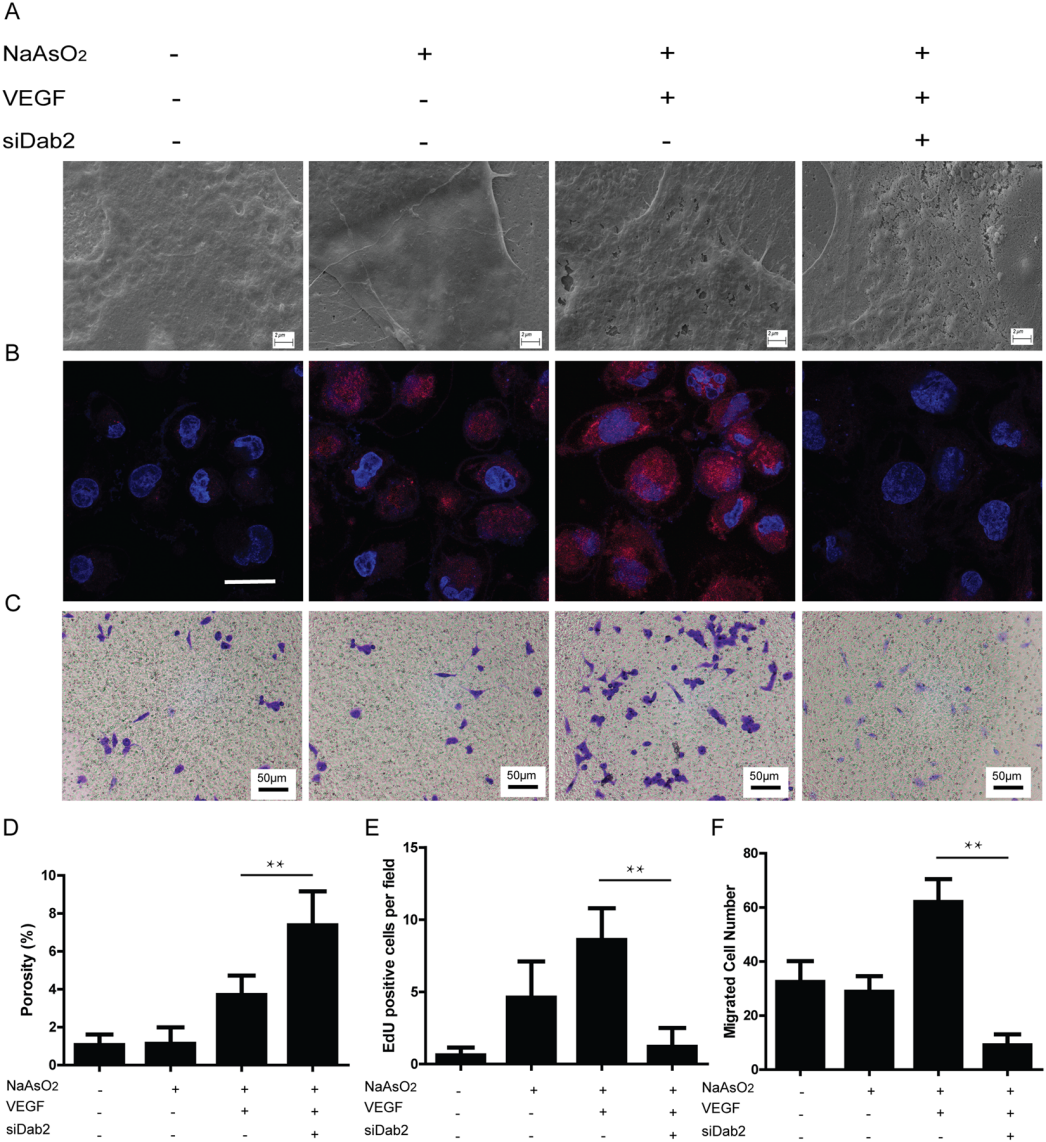
Involvement of Dab2 in VEGF receptor endocytosis, regulating LSEC dedifferentiation, proliferation and migration (**A**) Representative SEM images of fenestrae in SK-HEP1 from NaAsO2- VEGF- siDab2-, NaAsO2 + VEGF- siDab2-, NaAsO2 + VEGF + siDab2- and NaAsO2 + VEGF + siDab2 + group *in vitro*. Bar = 2μm. All experiments were repeated at least three times. (**B**) Representative EdU staining images in SK-HEP1 groups described above *in vitro*. Bar = 50μm. All experiments were repeated at least three times. (**C**) Representative crystal violet staining images of migrated cells in SK-HEP1 groups described above. Bar = 100μm. All experiments were repeated at least three times. (**D**) Quantitative porosity of fenestrae in SK-HEP1 groups described above *in vitro*. **P < 0.01. (**E**) Cell numbers per field of EdU stained SK-HEP1 groups described above *in vitro*. **P < 0.01. (**F**) Cell numbers per field of crystal violet stained SK-HEP1 groups described above *in vitro*. **P < 0.01.

To verify this hypothesis, we explored the role of Dab2 in VEGF receptor endocytosis and LSEC dedifferentiation in SK-HEP1 human liver sinusoidal endothelial cell line. SK-HEP1 cultured 3 days or treated with 5 μM NaAsO_2_ for 12 h can induce SK-HEP1 dedifferentiation (Fig. 4A,D). Western blot of surface VEGFR1 and VEGFR2 and immunofluorescence images showed that NaAsO_2_ might induce endocytosis of VEGFR1 and VEGFR2 and 40ng/ mL VEGF can enhance this response to maintain these receptors’ steady expression on the cell surface, regardless of their total expression alteration after NaAsO_2_ and VEGF induction, as these responses augmented VEGF-VEGFR signaling in perinuclear localization (Supplemental Fig. 3C,D). The biological significance of Dab2 in LSECs was evaluated by knockdown experiment using siRNA (Supplemental Fig. 3A), and the expression of Dab2 was decreased after siRNA transfection (Supplemental Fig. 3B). Dab2 knockdown significantly suppressed VEGFR1 and VEGFR2 endocytosis, increased their expression on cell surface and attenuated perinuclear VEGF signaling (Supplemental Fig. 3C,D). Meanwhile, fenestration of SK-HEP1 cells was well maintained in Dab2 knockdown plus VEGF group, compared with NC, Arsenic and Arsenic plus VEGF group (Fig. 4A,D), suggesting that Dab2 plays a key role in LSEC dedifferentiation. In addition, cell proliferation and migration, two key steps in angiogenesis, was also reduced after Dab2 knockdown (Fig. 4B,E and C,F), indicating that Dab2 inhibition may suppress LSEC dedifferentiation associated angiogenesis.

## Discussion

### The proteomic aspect of differentially expressed proteins between normal and dedifferentiated LSECs

LSECs are morphologically unique endothelial cells, also the only mammalian sinusoidal endothelium combining non-diaphragm fenestrae without basement membrane^22,23^. They are also functionally unique, providing high rate and capacity to clear waste from the circulation^24^, and LSECs are the initial target of chemical liver injury^25^ and susceptible to ischemia-reperfusion injury^26^. In addition, LSECs maintain HSC quiescence, inhibiting intrahepatic vasoconstriction and liver fibrosis development^4,27^. LSEC dedifferentiation occurs following liver injury in animal models and patients^5,6^, then LSECs lose their protective properties and promote liver injury associated angiogenesis and vasoconstriction. LSEC dedifferentiation is an early event preceding HSC activation and the onset of liver fibrosis. Therefore, it could be a preliminary step necessary for liver fibrosis. Although some researchers have illustrated multiple mechanisms of LSEC dedifferentiation, the extensive and systematic analysis of proteome between normal and dedifferentiated LSECs has not been identified. NaAsO_2_ was reported by Straub *et al*. to cause functional changes in signaling of LSEC dedifferentiation, mainly pathogenesis of intrahepatic vascular diseases^8,9^. Therefore, availability of NaAsO_2_-induced liver chronic injury model *in vivo* and LSEC-resolved proteome could help to understand the global protein changes during LSEC dedifferentiation in liver disease associated angiogenesis. The SK-HEP1 cell line was proved by Heffelfinger *et al*. as human endothelial origin^28^. Cogger *et al*. further discovered theis cell line displayed many characteristics of LSECs such as fenestration, uptake of Ac-LDL and tube forming, making it more appropriate for specific LSEC research^29^. Therefore, we applied SK-HEP1 to analyze the biological effects of candidate proteins.

To our knowledge, this is the first comprehensive proteomic analysis of LSEC dedifferentiation. The present iTRAQ-based proteomic study identified a variety of novel proteins associated with LSEC dedifferentiation and dysfunction, extending our understanding of this process. Based on GO and DAVID analysis, the most enriched biological function categories of up-regulated proteins in dedifferentiated LSECs were nucleotide, organic acid metabolism, oxidative stress, small molecular and lipid metabolism, cell death regulation and endocytosis. Most function categories have been reported to associate with LSEC dedifferentiation^8,9,30,31^, except for receptor endocytosis. Therefore, CLTC, which is an important component of clathrin-coated vesicle^32^ and participate in endocytosis, was screened for further validation by western blot and immunohischemistry in our study. Our results supported that CLTC may be a potential marker of endocytosis during LSEC dedifferentiation. Instead, the top enriched biological function categories of down-regulated proteins in LSEC dedifferentiation were transcription regulation, actin cytoskeleton reorganization, cell migration, immune system process, ribosome biogenesis, apoptotic process, angiogenesis, glycerophospholipid metabolism and cellular lipid metabolism. Several proteins involved in innate immune response were simultaneously down-regulated, including Galectin-3, SAMHD1 and Rab-10, determined by proteomic analysis and western blot validation, suggesting that reduced innate immune response caused defense line damage against injury insult, leading to the initiation of liver diseases.

### The role of Dab2 in LSEC dedifferentiation

The expression of Dab2, a clathrin-associated sorting protein, was induced during LSEC dedifferentiation based on our proteomic data. As described previously, Dab2 is referred as a mediator of VEGF receptor endocytosis, leading to angiogenesis^13,33^. We supposed that Dab2 contributed to LSEC dedifferentiation through VEGF receptor endocytosis. With our results, Dab2 expression was increased by NaAsO_2_ administration, resulting in VEGFR1 and VEGFR2 endocytosis and localization at the perinuclear region, and in addition, VEGF promoted these responses, furthermore augmented its intracellular signaling. Consistently, Dab2 knockdown reduced VEGFR1 and VEGFR2 endocytosis and declined VEGF signal in perinuclear, which resulted in the maintenance of LSEC fenestration and limited LSEC proliferation and migration rate to inhibit angiogenesis process, suggesting that Dab2 knockdown may contribute to anti- LSEC dysfunction and anti- angiogenesis therapy. It is reported that VEGF maintains LSEC differentiation and prevents liver fibrosis progression^4,27^, but it is confused that VEGF also induces angiogenesis, which may lead to liver disease progression^34,35^, but so far, this contradiction has not yet been clearly clarified. In this study, we supposed that the effects of VEGF on LSECs depended on the receptor endocytosis instead of VEGF concentration. Dab2, regulating clathrin-coated receptor endocytosis, induced VEGFR1 and VEGFR2 endocytosis, and enhanced VEGF signaling which has been linked to angiogenesis. Therefore, Dab2 may be a mediator initiating LSEC dedifferentiation and multiple liver injury process. Targeting drugs inhibiting Dab2 expression might provide a novel therapy that improves LSEC homeostasis and blocks angiogenesis associated with multiple liver diseases.

In brief, proteome changes between normal and dedifferentiated LSECs using iTRAQ provided the comprehensive database of differentially expressed proteins. Bioinformatic analysis of proteome data promoted our understanding of the characteristics of dedifferentiated LSECs, such as receptor endocytosis, multiple compound metabolisms, and reduced innate immune response. Moreover, this study revealed the role of Dab2 in VEGF receptor endocytosis and provided insight into LSEC dedifferentiation and angiogenesis. In conclusion, as the first comprehensive proteomic analysis of dedifferentiated LSECs, the data provided here will enhance our understanding of LSEC effects on liver injury and concomitant angiogenesis, and will accelerate the development of diagnostic and therapeutic strategies for multiple liver diseases.

## Materials and Methods

### Animal studies

Mice were housed at the Institutional Animal Care Facility of Beijing Proteome Research Center. All experiments were performed in accordance with relevant guidelines and regulations for laboratory animals. 6-8 week old male C57BL/6J mice (purchased from Vital River Co, Beijing, China) were used for NaAsO_2_-induced LSEC dedifferentiation and CCl_4-_induced chronic liver injury model. The animal use protocol was approved by the Animal Care Committee of Beijing Proteome Research Center. For NaAsO_2_-induced LSEC dedifferentiation model, standard mouse chow and drinking water solutions were fed freely for 5 weeks to normal control mice (n = 12) housed for three per box. Fresh drinking water solutions with 250 ng/ mL NaAsO_2_ and standard mouse chow were prepared 3 times per week using commercially bottled drinking water for arsenic-exposed mice (n = 12), as described previously^9^. For CCl_4_-induced chronic liver injury mouse model (n = 12), CCl_4_ in olive oil was intraperitoneal administration twice per week for 6 weeks, mice administrated with olive oil was referred as normal controls (n = 12), according to a previous study^36^.

### Cell isolation and culture

LSECs were isolated from male C57BL/6 J mice via protocols adapted from modified method^14,15^. Briefly, after mice anaesthetized by pelltobarbitalum natricum, the liver was perfused *in situ* with two steps of Hanks buffer and collagenase solution, respectively, and then excised and digested in perfusion buffer. The resulting supernatant was centrifuged at 50 g for 3 min to eliminate hepatocytes. The resting supernatant enriched in HSCs, KCs and LSECs was separated by Optiprep^TM^ density gradient medium. The cell fraction between the 8.2 and 17.6% Optiprep^TM^ enriched in LSECs and KCs was further separated by mouse LSEC binding magnetic beads (Miltenyi, Germany). Purity and viability of isolated LSECs were confirmed by CD146 + F4/80- and 7-AAD + flow cytometry, respectively, and quality of LSECs was detected by fluorescently labeled Ac-LDL.

Human LSEC line SK-HEP1 was purchased from American Type Culture Collection (Manassas, VA). Cells were cultured in 24-well plates with DMEM (Hyclone, South Logan, UT) supplemented with 10% fetal bovine serum (FBS) (Gibco, Grand Island, NY) and antibiotics.

### Differential proteome analysis based on iTRAQ labeling and Triple TOF MS

A total of 100 μg proteins of primary LSECs in normal and LSEC dedifferentiated samples were labeled with iTRAQ according to the Applied Bio systems iTRAQ labeling protocol (Foster city, CA). The digested peptides of each sample were labeled with 115 (normal LSECs) and 116 (dedifferentiated LSECs) iTRAQ reagents. The labeled peptides were mixed together, and then cleaned up the excess trypsin and iTRAQ reagents by Hamilton PRP™-C18 reversed phase HPLC column (Reno, NV) using Thermo DINOEX Ultimate 3000 BioRS system high-performance liquid chromatography (HPLC) (Grand Island, NY) before mass spectrometry. Replicate analyses were performed using 116 (normal LSECs) and 115 (dedifferentiated LSECs) iTRAQ reagents.

Mass spectrometric analysis was performed with an AB SCIEX Triple TOF 5600 System (Concord, Canada). Samples were chromatographed by a 90 min gradient from 2-30% (mobile phase A 0.1% (v/v) formic acid, 5% (v/v) acetonitrile; mobile phase B 0.1% (v/v) formic acid, 95% (v/v) acetonitrile) injected onto a 20μm Pico Frit emitter (New Objective) packed to 12 cm with Magic C18 AQ 3μm 120 Å stationary phase. MS1 spectra were collected in the range 350-1500 m/z for 250ms. The 20 most intense precursors with charge state 2-5 were selected for fragmentation, and MS2 spectra were collected in the range 50-2000 m/z for 100ms; precursor ions were excluded from reselection for 15 s.

In this study, the original MS/MS file data was analyzed by Protein Pilot software (version 4.0, AB SCIEX), the following parameters were used: the instrument was Triple TOF 5600, iTRAQ quantification, cysteine modified with iodoacetamide; biological modifications were selected as ID focus, the Quantitate, and trypsin digestion. Bias Correction and Background Correction was checked for protein quantification and normalization. Proteins with at least 95% confidence determined by Protein Pilot Unused scores (≥1.3) were reported, and the false discovery rate (FDR) was calculated and set up less than 1%. Fold changes ≥1.5 or ≤0.67 were considered significant.

### Bioinformatic Analysis of Differential Proteins

The bioinformatic analysis of differential proteins was performed with Gene Ontology Terms (http://www.geneontology.org/) and DAVID Annotation (https://david.ncifcrf.gov/). The lists of differential proteins were input into these platforms for identification of Subcellular localization and biological functional distribution. The false discovery rate was set less than 0.05.

### Statistical Analysis

The sample size (n) of each experimental group is described in each corresponding Figure legends, and all experiments were repeated at least three times. Data was expressed as the mean ± standard error with at least 3 independent experiments. To compare values between groups, the ANOVA or Student’s t test was used. *P* value < 0.05 was considered significant.

### Data availability statement

All data generated or analysed during this study are included in this published article and its Supplementary Information files.

### Ethical approval

All animal experiments were reviewed and approved by the Animal Care and Use Committee at the Academy of Military Medical Sciences to ensure the ethical and humane treatment of the animals.

## Electronic supplementary material

Supplementary Information

Supplementary Dataset 1

Supplementary Dataset 2

Supplementary Dataset 3
