# Supplementary material for: Proteomic Analysis Reveals Dab2 Mediated Receptor Endocytosis Promotes Liver Sinusoidal Endothelial Cell Dedifferentiation
Source: Sci Rep. 2017 Oct 18;7:13456. doi: 10.1038/s41598-017-13917-9 (PMC5647404; doi:10.1038/s41598-017-13917-9)

**Proteomic Analysis Reveals Dab2 Mediated Receptor Endocytosis Promotes Liver Sinusoidal Endothelial Cell Dedifferentiation**

**Author Names:**

Yuanxiang Lao ^1,2, #^, yx_lao@126.com

Yanyan Li ^3, #^, angelina5655@126.com

Yufang Hou^2^, verahou606452@163.com

Huahai Chen^2^, chenhuahai2008@163.com

Bintao Qiu ^2^, [qiubintao-003@163.com](mailto:qiubintao-003@163.com)

Weiran Lin ^2^, laop_530@163.com

Aihua Sun^2^, sunah620@126.com

Handong Wei ^2^, weihd@163.com

Ying Jiang ^2,*^, jiangying304@hotmail.com

Fuchu He ^1, 2,*^ hefc@bmi.ac.cn

^1^Institute of Basic Medical Sciences, Chinese Academy of Medical Sciences, School of Basic Medicine, Peking Union Medical College, Beijing, China

^2^State Key Laboratory of Proteomics, National Center for Protein Sciences, Beijing, Beijing Proteome Research Center, Beijing Institute of Radiation Medicine, Beijing, China

^3^School of Life Sciences, Tsinghua University, Beijing, China

^*^Corresponding authors.

**Supplemental Materials and Methods**

**Reagent information**

Masson's trichrome staining kit was purchased from Leagene Biotech (Beijing, China). Ac-LDL was purchased from Invitrogen (Carlsbad, CA). WB secondary antibodies: goat anti rabbit, goat anti mouse were purchased from Cwbiotech (Beijing, China). WB primary antibodies: CD31, Caveolin-1, Rac1 were purchased from Abcam (Cambridge, UK); CLTC, Dab2, SAMHD1, Galectin-3, Rab-10 were purchased from proteintech (Rosemont, IL). Immunofluorescence secondary antibodies: goat anti rabbit Alexa@488 and TRITC secondary antibodies were purchased from Abcam (Cambridge, UK). Immunofluorescence primary antibodies: VEGFR1, VEGFR2 and VEGF-A were purchased from proteintech (Rosemont, IL). Phalloidin was purchased from Invitrogen (Carlsbad, CA).

**Electron microscopy and quantitative imaging**

For scanning electron microscopy (SEM), cultured SK-HEP1 cells and liver tissues were fixed with glutaraldehyde, postfixed with OsO_4_, dehydrated with graded alcohols, dried with hexamethyl disilazane, sputter-coated with gold and examined using a FEI Quanta 200 scanning electron microscope (FEI, Hillsboro, OR). For transmission electron microscopy (TEM), isolated LSEC sediment was fixed with 4% paraformaldehyde, then was immersion fixed in 2% glutaraldehyde, postfixed in 1% osmium tetroxide, dehydrated in a graded series of ethanol, and embedded in Epon. Ultra-thin sections (80 nm) were cut on an Ultra microtome EM UC6 (Leica, Vienna, Austria), stained with 1% uranyl acetate, counterstained using the Reynolds method, and examined on an H-7650B electron microscope (Hitachi).

Porosity (percentage of LSEC surface occupied by fenestrae) was measured in SEM and TEM micrographs of cells, in brief, total LSEC surface area and the open area of individual fenestrae were quantified. Open areas were summed, divided by total surface area, expressed as a percentage of open area, and averaged. Each average was taken from 15 images and analysis was done using ImageJ software (https://imagej.nih.gov/ij/).

**Western blot analysis**

For total protein western blot, cell samples were lysed in RIPA buffer with protease inhibitors. After 30 minutes’ standing, proteins were fully extracted from lysate using a scroll oscillator or a tissue homogenizer. And then 12000g centrifugation was performed to collect supernatant. For surface protein western blot, receptors were labeled with 0.5mg/ml sulpho-NHS-biotin (Abcam) according to the manufacturer's instructions. Excess biotin was quenched by 100mM glycine in PBS, the cells were dissolved in 0.8ml lysis buffer (25mM Tris-HCl at pH 7.5, 150mM NaCl,5mM EDTA-NaOH at pH 8.5, 0.5% Triton-X100, 0.5% NP-40, 100mM NaF, 10mM Na_4_P_2_O_7_, 1mM Na_3_VO_4_ and cocktail protease inhibitor (Roche, 1:50). The lysates were precipitated with streptavidin agarose beads (Invitrogen). Followed by protein quantification using BCA protein assay kit or gel electrophoresis, equal amount of sample protein was loaded on 10% or 12% SDS-PAGE gels and transferred to NC membranes using an electro blotting apparatus. The blots were blocked in skimmed milk buffer and next incubated with detected antibodies overnight at 4℃. The blots were incubated with the corresponding secondary antibodies followed by membrane wash for 5 minutes and three times. With the help of DAB horseradish peroxidase color development kit, the bands were visualized and quantified by Image J software.

**Immunochemistry analysis**

For immunocytochemistry, HSCs and LSECs were cultured on coverslips and fixed with 4% formaldehyde for 30 minutes at room temperature. And then permeated with 1% Triton X-100 and blocked with 3% Bovine albumin (BSA), HSCs and LSECs were incubated with primary antibodies. After washing three times with PBS, all slices were followed by anti-rabbit Alexa488 or TRITC conjugated secondary antibodies. Slices were observed using a Zeiss LSM 810 confocal microscope (Zeiss Co. Germany).

For immunohistochemistry, paraffin-embedded liver samples were sectioned, dewaxed and hydrated, and subsequently incubated for 10 minutes in 3% hydrogen peroxide to block endogenous peroxidase. Antigen retrieval was performed by heating in 10 mmol/L sodium citrate buffer (pH 6.0) for 10 minutes. Sections were blocked in 3% BSA for 30 minutes and incubated with primary antibodies. Control sections were incubated overnight at 4 ℃in each corresponding serum for primary antibody sources. Polymer–horseradish peroxidase antibody was used as secondary antibody. The 3, 3'-Diaminobenzidine (DAB) substrate was used in the detection procedure. Slices were observed using a Nikon LV100D microscope (Nikon Co., Japan).

**Small Interfering RNA Transfection, LSEC dedifferentiation and proliferation analysis**

The siRNA sequences specific for Dab2, as well as the control siRNA, were purchased from Genepharm. Transfection. The sense sequence of Dab2 siRNA negative control was (5’-3’) UUC UCC GAA CGU GUC ACG UTT, and antisense (5’-3’) ACG UGA CAC GUU CGG AGA ATT. The sense sequence of the first Dab2 siRNA was (5’-3’) GUC CAA CAG AAA GCA AAG ATT, and antisense (5’-3’) UCU UUG CUU UCU GUU GGA CTT. The sense sequence of the second Dab2 siRNA was (5’-3’) CCA GCA GUG AGA ACU CAA ATT, and antisense (5’-3’) UUU GAG UUC UCA CUG CUG GTT. The sense sequence of the third Dab2 siRNA was (5’-3’) CCU GCC AGU UAC CAA AUC UTT, and antisense (5’-3’) AGA UUU GGU AAC UGG CAG GTT. The transfection experiment was performed according to the manufacturer’s recommendations using lipofactamine 2000 CD reagent (Invitrogen, Carlsbad, CA). After 48 h transfection, the efficiency of siRNA-mediated mRNA and protein degradation was assessed by quantitative real-time polymerase chain reaction (qRT-PCR). The effects of Dab2 knockdown on LSEC dedifferentiation and proliferation were measured by scanning electron microscope analysis of fenestration and cell counting under the bright field of microscope. The effects of siRNA transfecteion on SK-HEP1 cells migration were measured by using a modified transwell chamber assay. Two days after transfection, 2 ×10^4^ cells in DMEM containing 0.5% FBS were plated on the upper chamber of each Transwell with 8μm pores (Costar, Corning Inc., NY), and the lower chamber was added with DMEM containing 2.5% FBS. Transfected cells were incubated for 24 h at 37°C in 5% CO_2_. Non-migrating cells were removed from the upper surface of the membrane with cotton swabs. Membranes were stained with crystal violet and mounted onto glass slides, and migration was quantified by counting cells in five fields.

**qRT-PCR analysis**

SK-HEP1 cells with transfection treatment by siRNA 1, 2, 3 and negative control were used to extract RNA by RNA extraction kit (CW Bio, China). The first step for reverse transcription contained template RNA, dNTP mix and nuclease-free water (Invitrogen, CA). The second step for PCR contained cDNA from the first step, sense and antisense primers, master mix and nuclease-free water (Invitrogen, CA). PCR was set for 30 cycles. And we can get the quantification results for each sample.

**EdU analysis**

Sk-HEP1 cells were incubated at 37°C for 2h in medium with 50μM EdU. For the in vivo EdU assay, cells were fixed with 4% formaldehyde for 30 min, incubated with glycine (2 mg/ml) for 5 min and treated with 0.5% Triton X-100 for 10 min to permeabilize cells. After being washed with phosphate-buffered saline, cells were incubated with Apollo reaction cocktail for 30 min and treated twice with 0.5% Triton X-100. DNA was stained with Hoechst 33342 stain for 30min and visualized with fluorescence microscopy. Five groups of confluent cells were randomly selected from each sample image.

**Transwell Migration assay**

Serum starved cells (25,000-50,000) in 0.1mL of FBS-free medium were seeded onto membrane inserts with 8μm pores (Corning), medium containing 2.5% FBS was added into the lower chamber and incubated overnight. Cells that had migrated through the matrigel were then fixed and stained with methylrosanilnium chloride solution. The membrane attached with migrated and invaded cells was placed on a glass slide and total cell numbers from five random fields under 20-fold magnifications were quantified with an Nikon LV-100D microscope and ImageJ software (https://imagej.nih.gov/ij/). All experiments were performed independently at least two times. Representative experiments are shown.

**Statistical Analysis**

Data was present as mean ± standard error with at least 3 independent experiments. To compare values between groups, the ANOVA or Student’s t test was used. *P* value <0.05 was considered significant.

**Supplemental Figures**

**
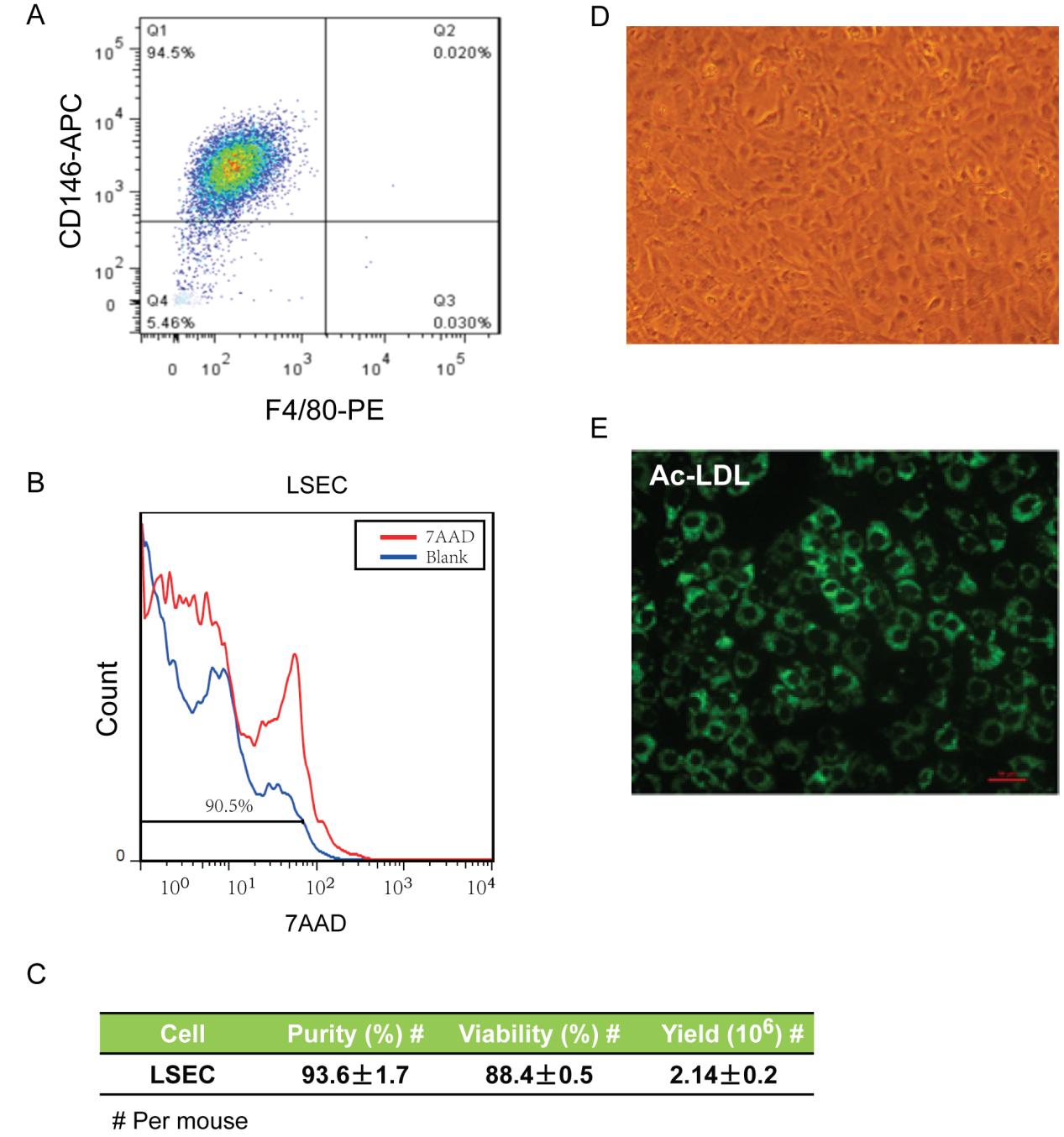
**

**Supplemental Figure 1 Purity and characterization of primary LSECs** (A) Purity and (B) viability of primary LSECs were analyzed by flow cytometry stained with CD146+F4/80- and 7-AAD+, respectively. (C) Statistical analysis of purity, viability and yields of primary LSECs. Data was expressed as mean ± standard error per mouse with at least 5 independent experiments. (D) Characterization of primary LSECs was examined by cobble stone like statue observed under bright field of microscope and (D) uptake of acetylated low density lipoprotein (Ac-LDL), Bar=50μm. All experiments were repeated at least three times.


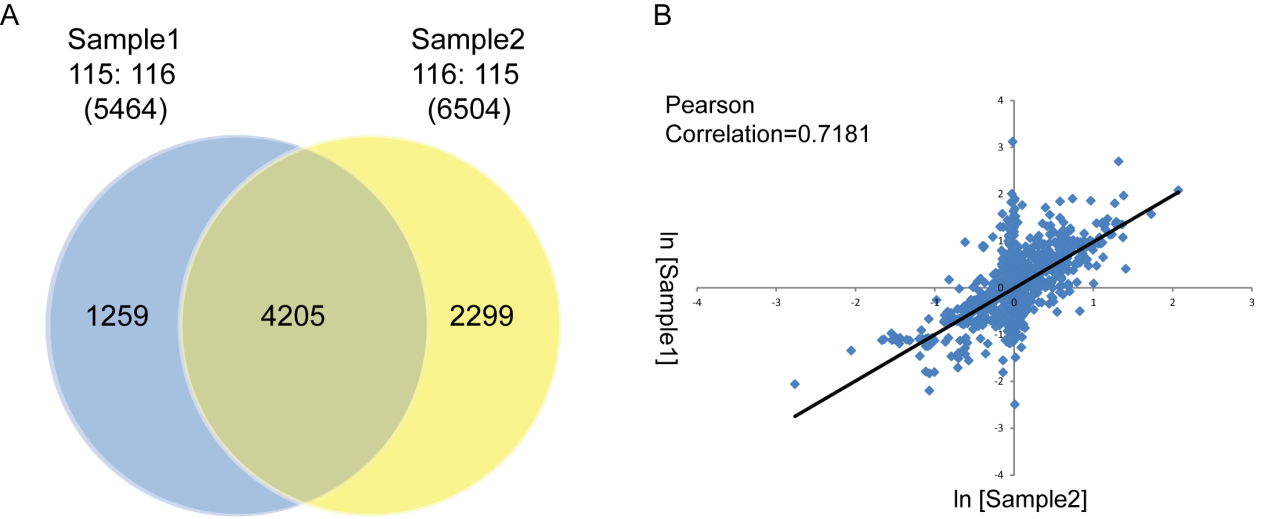


**Supplemental Figure 2** (A) Venn diagram depicting the overlap of proteins identified in two independent iTRAQ experiments. Numbers in brackets indicate the number of identified proteins for each sample. (B) To examine the biological reproducibility, linear regression analyses were performed on ln-transformed 115/116 or 116/115 ratios (Normal LSECs/dedifferentiated LSECs) of two independent analyses. Pearson correlation coefficient between sample 1 and 2 is 0.7181, *P*<0.0001.


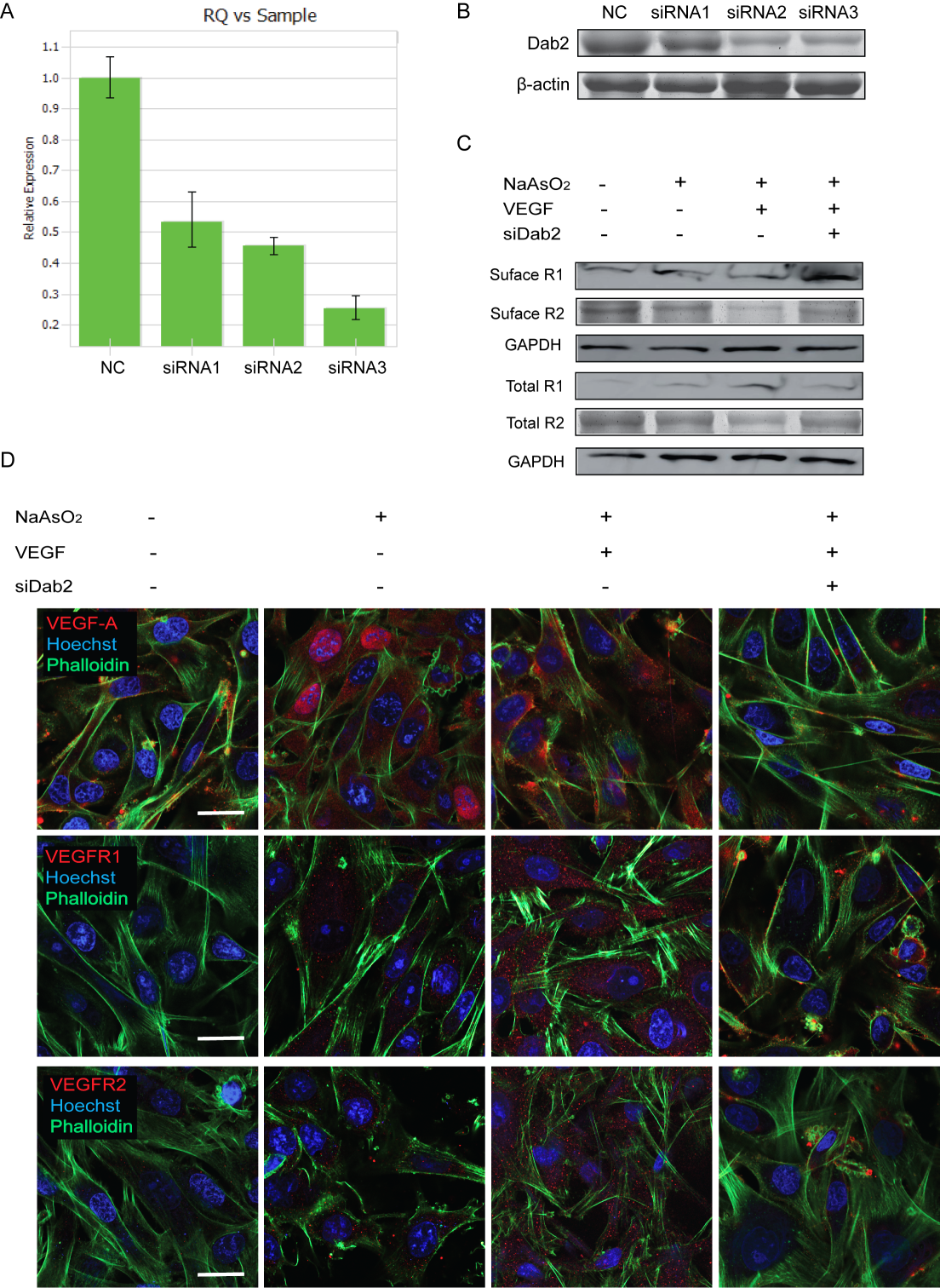


**Supplemental Figure 3 Knockdown efficiency of siRNAs** (A) SK-HEP1 cells were transfected with 3 Dab2 specific siRNA sequences or with NC siRNA. After 48 h, their mRNA and protein levels were determined by qRT-PCR and Western blot, while GAPDH is used as loading control, all experiments were repeated at least three times. (B) Dab2 protein levels for the above groups in SK-HEP1 were determined by western blot, β-actin is used as loading control, All experiments were repeated at least three times. Full-length blots are included in Supplemental Information. (C) Dab2 knockdown on VEGF receptors in SK-HEP1 from NaAsO2- VEGF- siDab2-, NaAsO2+ VEGF- siDab2-, NaAsO2+ VEGF+ siDab2- and NaAsO2+ VEGF+ siDab2+ group were determined by western blot, GAPDH is used as loading control, all experiments were repeated at least three times. Full-length blots are included in Supplemental Information. (D) Immunofluorescence of VEGF-A, VEGFR1 and VEGFR2 expression in SK-HEP1 groups in vitro. Bar=50μm. Nuclei: Hoechst 33342 (blue). All experiments were repeated at least three times.


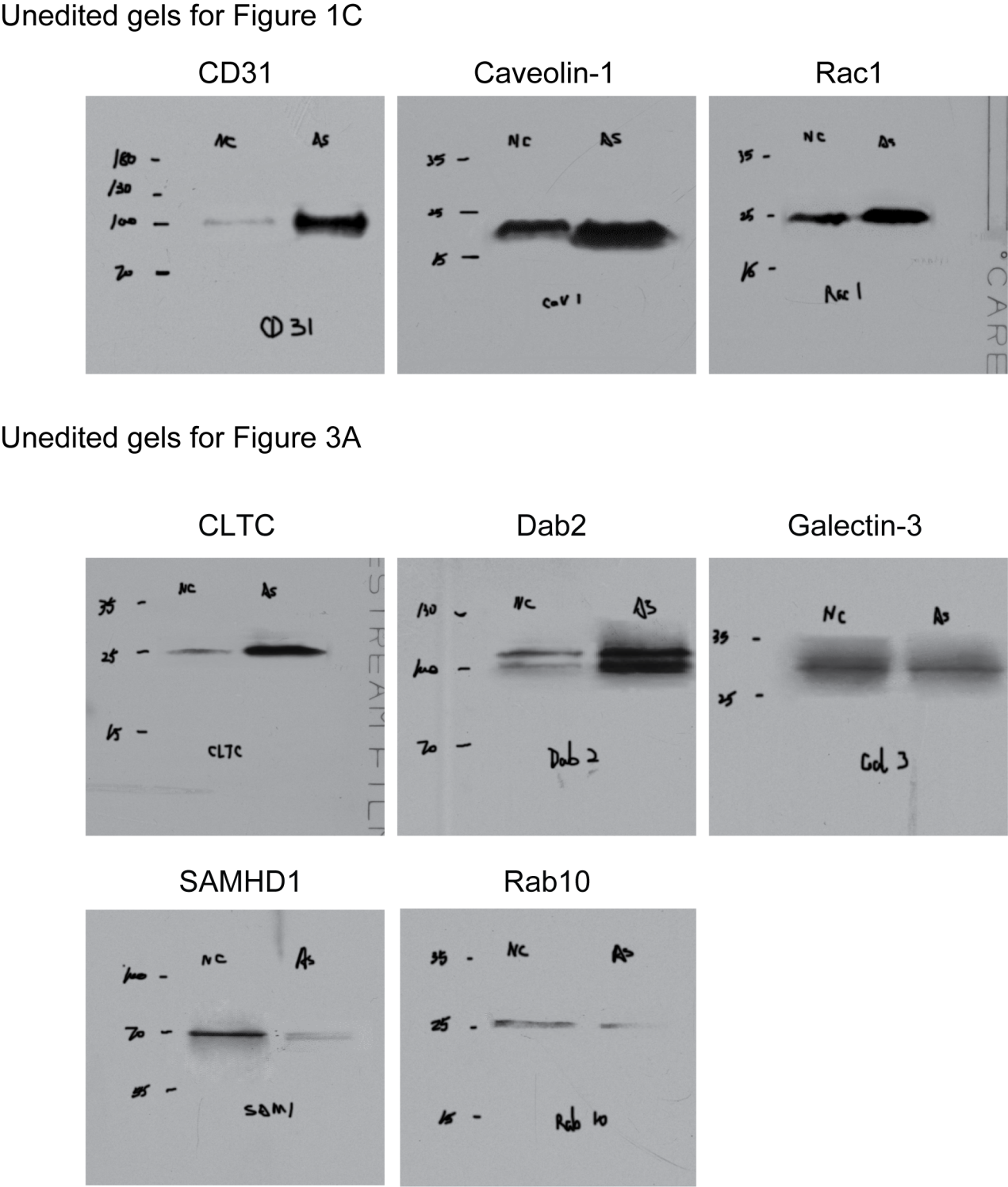


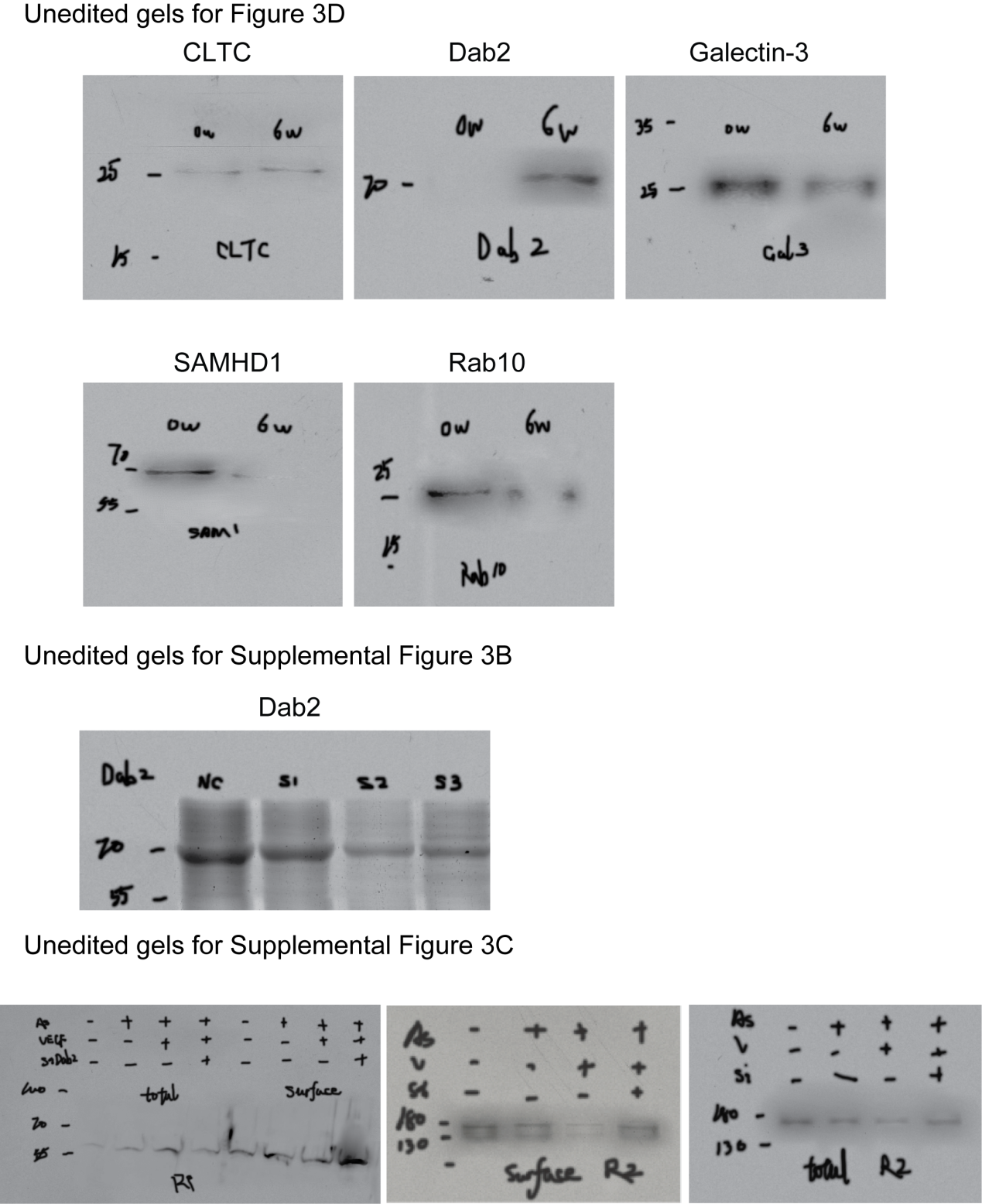

Supplement: Supplementary file 1 — Supplementary Information [file 41598_2017_13917_MOESM1_ESM.docx]
